# Environmental Association Identifies Candidates for Tolerance to Low Temperature and Drought

**DOI:** 10.1534/g3.119.400401

**Published:** 2019-08-22

**Authors:** Li Lei, Ana M. Poets, Chaochih Liu, Skylar R. Wyant, Paul J. Hoffman, Corey K. Carter, Brian G. Shaw, Xin Li, Gary J. Muehlbauer, Fumiaki Katagiri, Peter L. Morrell

**Affiliations:** * Department of Agronomy and Plant Genetics, University of Minnesota, St. Paul, MN 55108 and; † Department of Plant and Microbial Biology, Microbial and Plant Genomics Institute, University of Minnesota, St. Paul, Minnesota 55108

**Keywords:** cold, drought, adaptation, barley, allele frequency differentiation, mixed model association

## Abstract

Barley (*Hordeum vulgare* ssp. *vulgare*) is cultivated from the equator to the Arctic Circle. The wild progenitor species, *Hordeum vulgare ssp. spontaneum*, occupies a relatively narrow latitudinal range (∼30 - 40° N) primarily at low elevation (< 1,500 m). Adaptation to the range of cultivation has occurred over ∼8,000 years. The genetic basis of adaptation is amenable to study through environmental association. An advantage of environmental association in a well-characterized crop is that many loci that contribute to climatic adaptation and abiotic stress tolerance have already been identified. This provides the opportunity to determine if environmental association approaches effectively identify these loci of large effect. Using published genotyping from 7,864 SNPs in 803 barley landraces, we examined allele frequency differentiation across multiple partitions of the data and mixed model associations relative to bioclimatic variables. Using newly generated resequencing data from a subset of these landraces, we tested for linkage disequilibrium (LD) between SNPs queried in genotyping and SNPs in neighboring loci. Six loci previously reported to contribute to adaptive differences in flowering time and abiotic stress in barley and six loci previously identified in other plant species were identified in our analyses. In many cases, patterns of LD are consistent with the causative variant occurring in the immediate vicinity of the queried SNP. The identification of barley orthologs to well-characterized genes may provide a new understanding of the nature of adaptive variation and could permit a more targeted use of potentially adaptive variants in barley breeding and germplasm improvement.

When cultigens are disseminated from their region of origin, they must undergo adaptation to new climatic conditions (Gaut *et al.* 2018). These bouts of adaptation are most extreme for widely cultivated species such as barley and wheat, which are grown from the equator to the Arctic Circle. Efforts to identify the genetic basis of agronomic phenotypes in crops have typically started with a measurable phenotype or a proxy for the phenotype (*cf*. Doebley and Stec 1991). Correlations between the phenotype and genetic markers are identified using either quantitative trait locus (QTL) interval mapping in bi-parental populations (Lander and Botstein 1989) or association (also known as linkage disequilibrium or LD) mapping in a panel of unrelated individuals (Lander and Schork 1994). Both QTL and LD mapping have been identified as top-down approaches (Ross-Ibarra *et al.* 2007) because the investigator begins with a pre-identified phenotype and searches for marker correlations. Top-down approaches have proven extremely useful for identifying loci with a measurable effect on a phenotype, but the data collected generally do not aid in determining if the locus identified was important in the evolutionary origin of the trait. For example, in barley among 66 cloned genes, nearly half were identified by positional cloning (Hansson *et al.* 2018) and only one locus was initially identified as an allele frequency outlier (Comadran *et al.* 2012). Bottom-up approaches that identify genetic evidence of local adaptation or genomic signatures of selection have rarely been used to move from initial analysis to fully characterized genes (Doebley *et al.*, 2006; Ross-Ibarra *et al.*, 2007; Morrell *et al.*, 2012).

Genes identified in top-down approaches, by definition, contribute to measurable trait variation, but additional evidence is required to determine if the loci identified have played a role in adaptation (Ross-Ibarra *et al.* 2007; Kantar *et al.*, 2017). For example, the loss of inflorescence shattering in Asian rice was mapped to two loci (Konishi *et al.* 2006; Li *et al.* 2006). Only one locus, *qSH1*, shows evidence of selection in both the *japonica* and *indica* subspecies of rice, suggesting that only *qSH1* played a direct role in the domestication of both subspecies (Zhang *et al.* 2009).

Wild barley (*Hordeum vulgare* ssp. *spontaneum*), the progenitor of cultivated barley (*Hordeum vulgare* ssp. *vulgare*), occurs primarily within a limited latitudinal range of 30 - 40° N (Harlan and Zohary 1966). The geographic range of wild barley is bisected by the Zagros Mountains, with peaks up to 4,400 m, but wild barley occurs primarily at < 1,500 m (Zohary *et al.* 2012). Barley was domesticated from its wild progenitor ∼10,000 - 12,000 years ago. Domestication occurred at least twice (Morrell and Clegg 2007) and involved genetic contributions from across the geographic range of wild barley (Poets *et al.* 2015). The dissemination of cultivated barley beyond the initial centers of origin began ∼2,000 years after domestication (Willcox 2002). Barley landraces and modern cultivars are the result of pre-historic adaptation to growing conditions in Eurasia, North Africa, and much more recently, to Australia and the New World. In Eurasia, the process occurred as humans adopted an agropastoral lifestyle and spread from the Fertile Crescent into a variety of geographic regions. This included cultivation in regions with cooler and more mesic climates in Europe (Pinhasi *et al.* 2005) as well as drier climates in North Africa and Central Asia (Harris and Gosden 1996). Barley is frequently produced at high elevations in East Africa, Asia, and Europe and remains among the most important crops in Nepal and Tibet, where it is grown at elevations up to 4,700 m.

The adaptation of cereals such as barley and wheat to northern latitudes or dry climatic conditions involved changes in vernalization requirements (Yan *et al.* 2006; Dawson *et al.* 2015), growth habit (Turner *et al.* 2005; Zakhrabekova *et al.* 2012; Dawson *et al.* 2015), and flowering time (Comadran *et al.* 2012; Dawson *et al.* 2015). Wild species adapted to Mediterranean climates typically grow over winter and flower in the spring. This is known as a winter growth habit. Under cultivation, winter annuals such as barley and wheat have been adapted to colder climates through spring planting, known as a spring growth habit. Spring planting can make cultivation possible at higher latitudes but also increases exposure to frost and freezing conditions (Visioni *et al.* 2013).

Approaches for the identification of genetic variants contributing to environmental adaptation must discriminate between the effects of selection and neutral evolutionary processes (reviewed in Rellstab *et al.* 2015). Demographic effects acting on populations impact the entire genome, whereas selection alters allele frequencies at individual loci (Cavalli-Sforza 1966). The identification of loci involved in adaptation to the environment has involved measures that focus on differentiation in allele frequencies and approaches that identify correlations between allele frequencies and environmental variables (De Mita *et al.* 2013). Differentiation-based approaches were pioneered by Lewontin and Krakauer (1973) who proposed an approach to identify variants subject to differential selection between populations using allele frequency differences measured by F-statistics. F-statistic-based comparisons suffer from several weaknesses, including a high expected variance in *F*_ST_ values (Nei and Maruyama 1975) and the arbitrary nature of the partitioning of populations (Lotterhos and Whitlock 2014). If informative population partitions are defined, *F*_ST_ measures can identify loci subject to strong differential selection (Beaumont and Balding 2004). For examination of environmental adaptation, this approach partitions samples by categorical variables such as elevation and latitude and seeks to identify the largest differences in allele frequency among these partitions. It has the advantage of being applicable to samples of both populations and individual accessions (Rellstab *et al.* 2015).

In addition to differentiation-based approaches, the other broad class of approaches for identifying potentially environmentally adaptive variants focuses on correlations between genetic variants and environmental factors (De Mita *et al.* 2013). Correlative approaches have been developed that address population structure and relatedness among populations, thus better address heterogeneity of the environment than is possible with categorical comparisons (Coop *et al.* 2010). These approaches also take advantage of allele frequency estimates from each sampled population, ultimately determining whether the frequency of individual variants is more strongly correlated with environmental variables than with the underlying pattern of relatedness among populations (Coop *et al.* 2010; Günther and Coop 2013). The need for a sample of individuals from each population limits the application of this approach (Rellstab *et al.* 2015). Sampling design for studies seeking to identify the genetic basis of local adaptation is an active area of research (Lotterhos and Whitlock 2015). Mixed-model association using bioclimatic variables is an approach that can be appropriately applied to samples where a single individual represents each population (Eckert *et al.* 2010; Yoder *et al.* 2014; Rellstab *et al.* 2015). This approach explicitly controls for population structure with bioclimatic variables such as average temperatures and rainfall treated as “phenotypes” (Eckert *et al.* 2010; Yoder *et al.* 2014; Rellstab *et al.* 2015; Contreras‐Moreira *et al.* 2019).

Simulation studies suggest that the power to detect the genetic basis of adaptation using either differentiation or correlative approaches is dependent on factors such as the demographic history and migration model (De Mita *et al.* 2013; Lotterhos and Whitlock 2014), sampling design (Lotterhos and Whitlock 2015), mating system (De Mita *et al.* 2013; Lotterhos and Whitlock 2014), and whether variants that contribute to local adaptation are expected to demonstrate conditional neutrality or antagonistic pleiotropy (Tiffin and Ross-Ibarra 2014). In simulations, genotype-environment correlation methods have substantially more power to detect selection than differentiation-based approaches but also have higher rates of false positives (De Mita *et al.* 2013). *F*_ST_ comparisons have a low false positive rate but also generally lower power to detect loci subject to selection (De Mita *et al.* 2013; Lotterhos and Whitlock 2015). De Mita *et al.* (2013) note that genotype-environment approaches had higher power under an island model of migration while *F*_ST_ approaches have higher power under isolation by distance.

Here, we make use of 803 barley accessions from a geographically diverse collection of barley landraces genotyped with the barley 9K Illumina Infinium iSelect Custom Genotyping BeadChip (Comadran *et al.* 2012). The dataset includes all Old World landrace accessions from the USDA-ARS National Small Grains Collection (NSGC) Core Collection (Muñoz-Amatriaín *et al.* 2014) with a single accession representing each geographic location. The core collection was designed to maximize geographic coverage, which limited our analysis options. Here, we present *F*_ST_ outlier and mixed-model association analyses to identify loci potentially involved in cold and drought tolerance. As in a number of previous empirical studies, we make use of both differentiation and correlation-based analyses (Fang *et al.* 2012; Pyhäjärvi *et al.* 2013; Anderson *et al.* 2016) reviewed in Bragg *et al.* (2015). For differentiation analysis using *F*_ST_ comparisons, we focus on partitions of the sample that distinguish unique growth conditions. These include latitude, elevation, and spring *vs.* winter growth habit. To identify the factors that contribute most to allele frequency differentiation, we also calculated *F*_ST_ for a longitudinal comparison, a contrast reported in previous studies (Morrell and Clegg 2007; Saisho and Purugganan 2007; Poets *et al.* 2015). We address the following questions: 1) Which of the comparisons explains the largest portion of allele frequency differentiation? 2) How many previously reported cold temperature and drought tolerance related loci show evidence of contributing to climatic adaptation? 3) Do barley orthologs for genes associated with adaptive phenotypes show evidence of contribution to environmental adaptation in our sample? 4) Given the LD expected in a self-fertilizing species, how frequently are SNPs identified in our analyses in the proximity of potentially causative loci? For this final question, we make use of exome capture resequencing from a sample of 62 landraces drawn from the larger panel. This permits a direct estimate of LD between SNPs identified in our broader panel of accessions and variants in a window surrounding each locus.

We identified six barley genes previously reported to be involved in either cold or drought tolerance, or in flowering time. Furthermore, our analyses identified six additional barley orthologs of genes characterized as contributing to these traits in other plant species. A slight relaxation of the empirical cutoff for outlier *F*_ST_ values identified an additional four characterized barley genes and six orthologs from other plants. Considering both allele frequency outlier and bioclimatic association analyses, we identified 282 barley genes not previously reported to be associated with environmental adaptation. Comparisons of LD between SNPs in genotyping and resequencing suggest that roughly a quarter of the genes we identified on the basis of SNP genotyping are strong candidates for association due to the relatively low gene density in barley.

## Materials and methods

### Plant materials

We used 803 accessions of barley identified as landraces based on passport data from a core collection within the United States Department of Agriculture, National Small Grain Collection (Muñoz-Amatriaín *et al.* 2014). The 803 individuals were collected from Europe, Asia, and North Africa. These cover the range of barley cultivation in human pre-history (Willcox 2002; Pinhasi *et al.* 2005; Poets *et al.* 2015). Barley growth habits describe planting times. Spring growth habit is most common and constitutes 617 (76.8%) accessions in our sample. The balance of the sample includes 142 (17.7%) winter accessions, 16 (2.0%) facultative accessions that can be planted for spring or winter growth, and 28 accessions (3.5%) of unknown growth habit. Barley can also be divided into the ancestral two-row inflorescence type and the denser six-row type. Our sample includes 542 (67.5%) accessions of six-row barley, 219 (27.3%) two-row accessions, with the remaining 42 (5.2%) accessions of unknown row type. The reported geographic coordinates for each accession were manually confirmed to identify potentially inaccurate locations, and landraces with highly doubtful locations were filtered out (Table S1). The elevations of collection locations were inferred from the NASA Shuttle Radar Topographic Mission (SRTM) 90 m data (http://www.cgiar-csi.org/) on Oct 7, 2015 using the getData function from R package ‘raster’ (Hijmans *et al.* 2016).

### Genotyping data

All samples were genotyped using the 7,864 SNPs on the 9K Illumina Infinium iSelect Custom Genotyping BeadChip (Comadran *et al.* 2012). The genotyped data were published in Muñoz-Amatriaín *et al.* (2014). The SNPs are distributed across the seven chromosomes of the diploid barley genome. Because of the relatively large size of the barley genome, the SNP panel includes ∼1 SNP per 648 Kb in the 5.1 Gb genome (The International Barley Genome Sequencing Consortium 2012). For more details on the SNP discovery panel see the description in Comadran *et al.* (2012; 2015). Cultivated barley is 99% self-fertilizing (Wagner and Allard 1991; Bothmer 1992), and thus the number of unique chromosomes sampled is roughly equal to the sample size. The genotyped dataset was filtered for monomorphic SNPs and SNPs with > 20% missingness (Supplemental dataset 1). We culled SNPs in complete LD for comparative analyses, maintaining SNPs with lower missingness (Supplemental dataset 2).

### Estimating crossover relative to physical distance

We identified the physical position of 9K SNPs relative to the barley reference genome (Mascher *et al.* 2017) (Supplemental dataset 3; File S1). The crossover rate in cM/Mb was estimated using SNP physical positions relative to genetic map positions (Muñoz-Amatriaín *et al.* 2011). A Python script for this calculation and an R script for locally weighted scatterplot smoothing (LOESS) reported by Kono *et al.* (2018) are included in the project repository https://github.com/MorrellLAB/Env_Assoc.

### Sample differentiation

We estimated the degree of differentiation among individuals by principal components analysis (PCA). PCA was performed using the SmartPCA program from the EIGENSOFT package (Patterson *et al.* 2006) with SNP data converted from VCF using PLINK 1.90 (Chang *et al.* 2015).

### Exome resequencing data

We generated exome resequencing from 62 landrace accessions from a randomly chosen subset of landraces in the core collection. This includes 37 six-row spring and 25 two-row spring accessions (Table S2). DNA was extracted from leaf tissue collected from a single plant per accession at the 4-5 leaf stage using a standard 2X CTAB isolation protocol (Saghai-Maroof *et al.* 1984). The exome resequencing was performed using the NimbleGen exome capture design for barley (Mascher *et al.* 2017) followed by Illumina 125 bp paired-end resequencing at the University of Kansas Medical Center Genome Sequencing Facility, Kansas City, KS. The data were processed using publicly available software integrated with bash scripts in the ‘sequence_handling’ workflow (Hoffman *et al.* 2018). Details are in File S1. Variant calling is similar to that reported by Kono *et al.* (2016), with parameters specified in File S1.

### Heterozygosity, SNP diversity, and SNP annotation

Observed heterozygosity was calculated using PLINK 1.90 with the flag ‘–het’. The R package ape (Paradis *et al.* 2004) was used to calculate the average percent pairwise difference (Manhattan distance) between accessions. SNPs in coding and noncoding sequences and in amino acid changing positions within genes were identified using ANNOVAR (Wang *et al.* 2010) with gene models provided by Mascher *et al.* (2017) (Supplemental dataset 4).

### Bioclimatic and geographic variables

“WorldClim 1.4” bioclimatic data at a resolution of 2.5 min (Hijmans *et al.* 2005) were downloaded on Oct 7, 2015 using the getData ‘raster’ R function (Hijmans *et al.* 2016) in the R statistical language (R Core Team 2018). The latitude, longitude, elevation, and BIO1 to BIO19 values of the collection locations for each landrace are given in the phenotype data file (Supplemental data 5). Environmental variables can be divided into three categories, geographic factors, temperature, and precipitation. The latitude, longitude, and elevation were classified as geographic factors, BIO1 to BIO11 were classified as temperature, and BIO12 to 19 were classified as precipitation. To identify the relationship among the 22 variables given our sample locations, we performed independent components (ICs) analysis using the icaimax function from the ‘ica’ R package (Bell and Sejnowski 1995). ICs are conceptually similar to principal component summaries of data; however, we found that using the top three ICs appears to capture the cold temperature trend better than using the top three PCs (Table S3). Details of IC interpretation and comparison to Bioclim variables are reported in File S1.

### Environmental association mapping

To identify associations between genotypes and environmental variables, we used a compressed mixed linear model with a “Population Parameters Previously Determined” (P3D) algorithm (Zhang *et al.* 2010) implemented in the Genome Association and Prediction Integrated Tool (GAPIT) R package (Lipka *et al.* 2012). We used the genotyping data to infer the population structure by principal component analysis within GAPIT and used major principal components (Figure S1a) to control for structure in the mixed linear model. GAPIT implements kinship estimation from SNP data using the approach of VanRaden (2008). The kinship matrix was used in all mixed-model associations with environmental variables. We excluded SNPs with minor allele frequency (MAF) ≤ 0.01 from association analysis. We applied the Benjamini-Hochberg false discovery rate (FDR) correction. We report both adjusted *p*-values and FDR with an FDR threshold ≤ 0.25.

### F_ST_ estimation

To compare allele frequency differentiation in partitions of the data we calculated F-statistics (Wright 1949) for individual SNPs using the measure of Weir and Cockerham (1984) as implemented in the R package ‘HierFstat’ (de Meeûs and Goudet 2007). The *F*_ST_ analysis considered five partitions of the data, which were elevation, high latitude, low latitude, longitude, and growth habit.

The elevation comparison used a threshold of 3,000 m to delineate high elevation accessions. This includes accessions from the European Alps, the Caucasus, Himalayan, Hindu Kush Mountain regions, and the Ethiopian Plateau. Since wild barley typically grows below 1,500 m (Zohary *et al.* 2012), we also compared the allele frequency at three elevations: below 1,500 m, 1,500 m - 3,000 m, above 3,000 m.

We compared allele frequencies at two latitudinal ranges: (1) within the wild range of the species (30° N – 40° N) *vs.* landraces at latitudes higher than 40° N (high latitude), and (2) within the wild range of the species (30° N – 40° N) *vs.* landraces at latitudes lower than 30° N (low latitude). High latitude includes the northern extent of the range of wild barley and extends across Eurasia from the Central Iberian Peninsula to the Northern Japanese Archipelago. Low latitude includes the southern extent of the range of wild barley. For landraces, this extends from northwestern Africa to just south of the Japanese Archipelago. We also compared low and high latitude *vs.* the wild range in a single comparison.

For a longitudinal comparison, we divided the sample at 48° E, roughly through the Zagros Mountains, which coincides with the major axis of population structure in wild barley (Fang *et al.* 2014). The final comparison was spring *vs.* winter growth habit, with assignment based on USDA passport information.

To account for differences in sample size among partitions of the data (Table S4) (Bhatia *et al.* 2013), we used resampling with equalized sample numbers and 10 iterations without replacement. *F*_ST_ estimates for each SNP were averaged across 10 iterations and outliers were identified at the 99^th^ percentile of the distribution. To calculate the *p*-value for each *F*_ST_ value we performed 1,000 permutations. The details can be found in File S1.

### Identification of previously reported loci related to cold, drought tolerance, and flowering time

A literature search was used to identify genes previously reported to contribute to plant flowering time and cold or drought tolerance. Google Scholar searches were performed with the terms “cold OR freezing OR drought tolerance” or “flowering time” and “plant” or “barley” (Table S5-7). For each publication with these keywords in the title or abstract, we looked for evidence that individual genes were reported to contribute to flowering time, cold, or drought tolerance. The protein-coding sequences (CDS) of identified genes were used as the query or subject in BLASTN against the barley high-confidence CDS in May 2016 on the IPK Barley BLAST Server (Mascher *et al.* 2017). Barley genes and their interval information were extracted if the combined “Score,” “Identity,” “Percentage,” and “Expectation” produced the overall highest rank and the “Query length” was >100 bp. In the event of identical scores, all highest ranked hits were extracted.

The BEDOPS ‘closest-features’ function (Neph *et al.* 2012) was used to compare the locations of SNPs and gene intervals. Specifically, if the SNPs were located in the gene interval or 10 Kb up- or downstream of the closest genes, we considered those SNPs as identifying the closest gene.

### LD Around SNPs

For each 9K SNP identified in environmental association analysis or among *F*_ST_ outliers, we calculated LD with surrounding SNPs called from exome capture resequencing data. We focused on 200 Kb windows, 100 Kb upstream and downstream of the queried SNP. When the queried SNP was also genotyped by exome capture, this SNP was used for the LD analysis. If the queried SNP was not present in exome capture, we extracted the SNPs called from exome capture sequencing data surrounding the physical position of the queried SNP. Then we performed LD analysis using the proximate SNP with a MAF similar to the queried SNP. We filtered out SNPs using a MAF threshold of 1% for all of the SNPs called from the exome capture resequencing data. For LD analysis, filtering of variants could be anti-conservative, thus for this analysis, we removed SNPs with ≥ 50% missing data. We used the R package ‘LDheatmap’ (Shin *et al.* 2006) to calculate *r^2^* (Lewontin 1988).

### Inference of ancestral state

The ancestral state for each SNP from both 9K (Supplemental data 6) and resequencing datasets (Supplemental data 7) was inferred using whole-genome resequencing data from *Hordeum murinum* ssp. *glaucum* (Kono *et al.* 2018) with the programs ANGSD and ANGSD-wrapper (Korneliussen *et al.* 2014; Durvasula *et al.* 2016). We chose *H. murinum* ssp. *glaucum* for ancestral state inference because phylogenetic analyses have placed this diploid species in a clade relatively close to *H. vulgare* (Jakob *et al.* 2004). Previous comparison of Sanger and exome capture resequencing from the most closely related species, *H. bulbosum*, identified substantial shared polymorphism, resulting in ambiguous ancestral states (Morrell *et al.* 2013). Methods are detailed in Kono *et al.* (2018). Both minor and derived allele frequencies were calculated using a Python script.

### Haplotype analysis for individual genes

To assess evidence for functional diversity near SNPs identified in our analysis, we examined haplotype-level diversity in loci that flanked associations. We used exome capture resequencing from the panel described above. Overlapping SNP genotyping was extracted from SNP calls in a variant call format (VCF) file using ‘vcf-intersect‘ from vcflib (https://github.com/vcflib/vcflib). Missing genotypes were imputed using PHASE (Stephens *et al.* 2001; Stephens and Scheet 2005); PLINK 1.9 (Chang *et al.* 2015) was used to convert the VCF format into PHASE format. Homozygotes were treated as haploid and heterozygotes were treated as diploid samples for haplotype identification.

### Data availability

All sequences were submitted to the NCBI SRA associated with BioProject numbers PRJNA473780 and PRJNA488050. Supporting data (Supplemental data 1-10), including a VCF file of exome capture SNPs and the full results for environmental association and *F*_ST_, are available at https://doi.org/10.13020/adqb-bb41. Scripts for analysis and associated files are available on a project Github site at https://github.com/MorrellLAB/Env_Assoc. Supplemental material available at FigShare: https://doi.org/10.25387/g3.9478253.

## Results

### Summary of genotyping and resequencing data

We selected 803 barley landrace accessions that are a portion of the core collection within the United States Department of Agriculture, National Small Grain Collection (Muñoz-Amatriaín *et al.* 2014). All samples were genotyped using the 7,864 SNPs on the 9K Illumina Infinium iSelect Custom Genotyping BeadChip (Comadran *et al.* 2012) genotyping platform (Supplemental dataset 1, henceforth referred to as 9K SNPs). Genetic assignment identified four major groups of landraces previously identified by Poets *et al.* (2015) as Coastal Mediterranean, Central European, East African, and Asian (Figure S2). After quality filtering of the genotyping data and exclusion of landrace accessions without discrete locality information, our SNP genotyping dataset includes 5,800 SNPs in 784 accessions (Figure S3; Table S1; Supplemental dataset 2). Quality filtering of genotyping data resulted in the removal of 352 SNPs with > 20% missingness.

We also generated exome resequencing from 62 landrace accessions from a randomly chosen subset of the core collection. Resequencing and read mapping followed by read deduplication resulted in an average of 18X exome coverage for the sample. After variant calling and quality filtering, we identified 482,714 SNPs in 62 samples (Figure S3; Table S2; Supplemental dataset 7; Supplemental dataset 8, henceforth referred to as exome SNPs). The site frequency spectrums for SNPs in both the 9K and exome panels are shown in Figure S4. Average inbreeding coefficients estimated from SNP genotyping data and exome resequencing data are 0.996 (± 0.025) and 0.981 (± 0.008) respectively.

### Environmental association and F_ST_ outliers

**Figure 1:**
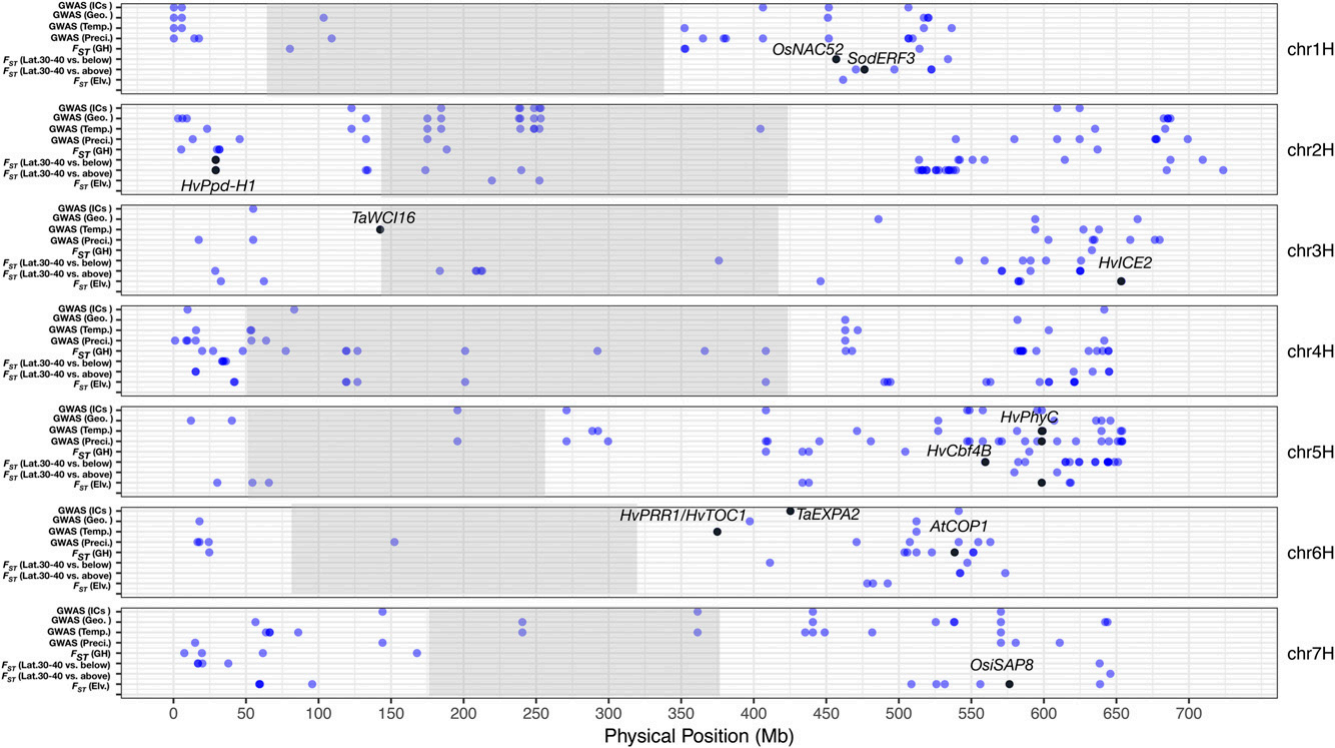
The genomic distribution of outlier SNPs identified in the *F*_ST_ comparisons of elevation (below 3,000m *vs.* above 3,000m), low latitude (below 30°N *vs.* 30-40°N), high latitude (30-40°N *vs.* above 40°N), growth habit (winter *vs.* spring), and association analysis of 21 bioclimatic variables, which are categorized into three classes (precipitation, temperature, and geographic variables) and ICs.

To examine associations between environmental variables and SNP diversity, we downloaded the latitude, longitude, elevation, and 19 bioclimatic variables of the collection locations for each landrace from “WorldClim” (Supplemental data 5). Because there is expected to be correlation among bioclimatic variables (Figure S5), we performed independent component analysis on the 19 variables to identify the subset of variables that best summarizes the range of environments occupied by barley landraces (Supplemental dataset 9). Combined with the 9K genotype data, we performed environmental association using a mixed linear model in GAPIT. The population structure and the kinship matrix were used in all mixed-model associations with environmental variables and ICs. Relatively few individuals showed close kinship; 95% of comparisons had pairwise distance > 0.1 based on the Manhattan distance between accessions (Figure S6). Initially, we identified 32 SNPs with the first three ICs with FDR ≤ 0.25. Loosening FDR to ≤ 0.3 or ≤ 0.4 identified an additional 45 SNPs or 77 in total. The first three ICs provide a limited summary of bioclimatic variation because they incorporate only eight bioclimatic variables (Table S3). The eight variables are not closely correlated to other bioclimatic variables (Figure S5). Limiting the analysis to ICs potentially excludes some of the bioclimatic signal associated with the remaining variables. Thus, we also examined each of the bioclimatic and geographic variables independently. The environmental association with bioclimatic and geographic variables and three ICs identified 155 SNPs in significant associations (with FDR ≤ 0.25) (Figure 1; Table S8).

**Figure 2:**
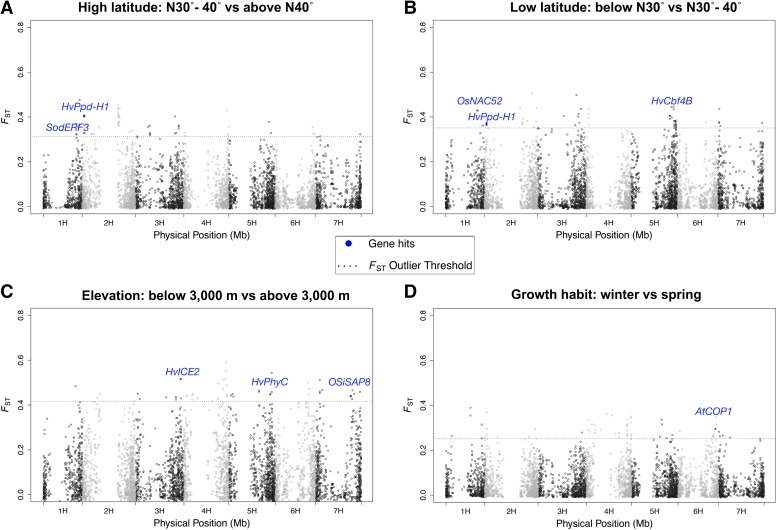
*F*_ST_ for 9K SNPs in samples from comparisons of (A) high latitude, (B) low latitude, (C) elevation, and (D) growth habit.

We examined allele frequency differentiation in five partitions of the data including elevation, high latitude, low latitude, longitude, and growth habit (Table S4). For both elevation and latitude, we calculated a single *F*_ST_ value with the samples divided into three groups (Table S9; Table S10; Figure S7). Since wild barley typically grows below 1,500 m (Zohary *et al.* 2012), the three groups of elevations were: below 1,500 m (low elevation), 1,500 m - 3,000 m (middle elevation), and above 3,000 m (high elevation). Since the geographic range of wild barley falls roughly between 30° N – 40° N, the three groups include: below 30° N (low latitude), 30° N – 40° N (middle latitude), and above 40° N (high latitude). We also calculated *F*_ST_ for low and middle elevations relative to high elevation, low to middle latitude, and middle to high latitude (Table S9; Table S10; Figure S7). While *F*_ST_ values for pairwise comparisons include many barley genes previously associated with adaptive phenotypes (see below), the outliers in the single elevation *F*_ST_ comparison did not include these candidate loci (Table S10-12). Thus, we focused on reporting outlier results on the two-level comparisons (Supplemental dataset 10). *F*_ST_ comparisons for elevation, latitude, and growth habit identified 203 outliers (using *F*_ST_ values in the upper 1% as the threshold) (Figure 1; Figure 2; Table S10). Considering both the environmental association and *F*_ST_ comparisons, we identified a total of 349 unique SNPs putatively associated with environmental adaptation in our genotyping panel (Figure 1).

Environmental associations and *F*_ST_ outliers shared nine SNPs in the coding portion of 11 annotated genes. The only characterized gene found in both analyses is *HvPhyC* in barley. For details regarding overlapping results see Table S11.

### Loci previously reported to contribute to environmental adaptation

Changes in flowering time and drought or cold tolerance are putatively adaptive traits for a cultivated species that has experienced a dramatic expansion in latitudinal range. Our results found four of the 57 genes previously identified in barley as contributing to flowering time, two of the 33 genes contributing to cold tolerance, and none of the 13 genes contributing to drought tolerance (Table 1; Table 3) with the *F*_ST_ threshold limited to the top 1% of values. However, we found six genes previously identified in barley as contributing to flowering time, four genes contributing to cold tolerance, and none of the 13 genes contributing to drought tolerance (Table 1), among the 2.5% of *F*_ST_ values.

**Table 1:** The number of barley genes detected with signals of adaptations and genotyped by 9K SNPs. The number in the parentheses is the fraction of total genes in that functional category that was genotyped or detected with signals of adaptation

	Total	Genotyped by ≥1 9K SNPs	Genotyped by ≥2 9K SNPs	With signals of adaptation
**Flowering time**	57	29 (50.9%)	9 (15.8%)	6 (10.5%)
**Cold tolerance**	33	20 (60.6%)	9 (27.3%)	4 (12.1%)
**Drought tolerance**	13	7 (53.9%)	3 (23.1%)	0 (0%)

The six loci, previously identified in barley and found to contribute to flowering time (using the upper 2.5% of *F*_ST_ values), include four loci identified as *F*_ST_ outliers. *HvPhyC* (Nishida *et al.* 2013) and *HvPpdH1* (Turner *et al.* 2005; Jones *et al.* 2008) occur among the upper 1% of *F*_ST_ values (Table 2; Table 3). *HvELF3 (Esp1L*/*eam8)* (Boden *et al.* 2014) and *HvPpd-H2* (*HvFT3*) (Casao *et al.* 2011) are included at the more liberal threshold of *F*_ST_ values in the upper 2.5% (Table 2). Environmental association identified two additional flowering time loci, *HvPRR1* (*HvTOC1*) (Ford *et al.* 2016) and *HvVrn-H1* (*HvAP1*) (Cockram *et al.* 2007), among the 155 outliers at FDR of 0.25 (Table 2).

**Table 2:** Loci identified in environmental associations or *F*_ST_ comparisons that were previously reported to contribute to flowering time, cold, and drought tolerance. Gene names are preceded by a two-letter prefix with the genus and specific epithet for the species where the gene was identified. This includes, *At* - *Arabidopsis thaliana*, *Ta - Triticum aestivum*, *Os - Oryza sativa*, *Br - Brassica napus*, and *Sod - Saccharum officinarum*. The *F*_ST_ comparisons involve the following: E: elevation; LL: Low Latitude; GH: growth habit; HL: high latitude. The * indicates that the gene was identified at the 97.5% threshold but not the 99% threshold

	Barley	Other plants	Bioclimatic variables	*F*_ST_
**Cold tolerance**	*HvCBF4B*	—	—	LL
	*HvDhn8	—	—	E
	*HvICE2*	—	—	E, GH
	*HvSS1	—	—	E
	—	*OsiSAP8*	—	E
	—	*TaWCI 16*	6	—
**Drought tolerance**	—	*AtACBP2	—	LL
	—	*AtIRX14	—	GH
	—	*AtABF3	—	HL
	—	*AtAREB1	—	HL
	—	*AtERECTA	—	HL
	—	*BrERF4	—	GH
	—	*SodERF3*	—	HL
	—	*TaEXPA2*	IC1	—
	—	*OsNAC52*	—	LL
**Flowering time**	—	*AtCOP1*	—	GH
	*HvELF3/Esp1L/eam8	—	—	HL, GH
	*HvPpd-H1/HvPRR37*	—	—	LL, HL
	*HvPpd-H2/HvFT3	—	—	GH
	*HvPhyC*	—	7, 14,17, & IC2	E
	*HvPRR1/HvTOC1*	—	8	—
	*HvVrn-H1/HvAP1*	—	3	—

Note: 1 = Annual mean temperature; 2 = Mean diurnal range (Mean of monthly (max temp - min temperature)); 3 = Isothermality (2/7) (* 100); 4 = Temperature seasonality (standard deviation *100); 5 = Max temperature of warmest month; 6 = Minimum temperature of coldest month; 7 = Temperature annual range (5 - 6); 8 = Mean temperature of wettest quarter; 9 = Mean temperature of driest quarter; 10 = Mean temperature of warmest quarter; 11 = Mean temperature of coldest quarter; 12 = Annual precipitation; 13 = Precipitation of wettest month; 14 = Precipitation of driest month; 15 = Precipitation seasonality (Coefficient of variation); 16 = Precipitation of wettest quarter; 17 = Precipitation of driest quarter; 18 = Precipitation of warmest quarter; 19 = Precipitation of coldest quarter; IC = Independent component.

**Table 3:** The number of SNPs identified by *F*_ST_ outlier approaches and the number of previously reported genes they identify. For each comparison, 55 SNPs in total were identified as outliers. Flowering time genes had one across all categories. Drought tolerance had zero SNPs detected in all categories. HL: high latitude; LL: Low Latitude; E: elevation; GH: growth habit

	Flowering time	Cold tolerance	
	SNPs	SNPs	Genes
**HL**	5	1	1
**LL**	4	2	2
**E**	1	1	1
**GH**	1	0	0

We also identified four loci previously reported as contributing to cold adaptation in barley, using *F*_ST_ values in the upper 2.5% (Table 3). This includes *HvCbf4B* (Stockinger *et al.* 2007) and *HvICE2* (Skinner *et al.* 2006) as *F*_ST_ outliers for the low latitude, elevation and growth habit comparisons at the top 1% threshold (Table 2). The upper 2.5% threshold for *F*_ST_ includes two additional characterized loci, *HvDhn8* (Choi *et al.* 1999) and *HvSS1* (Barrero-Sicilia *et al.* 2011) (Table 2).

A further six loci identified as *F*_ST_ outliers in the top 1% of values or in environmental associations in our barley panel had been previously identified as contributing to flowering time or cold or drought tolerance in other plant species (Table 2). This includes one flowering time-related locus characterized in *Arabidopsis thaliana*, *AtCOP1* (Xu *et al.* 2016), which was identified as an *F*_ST_ outlier in the top 1% of values. Two loci (*TaWCI16* and *OsiSAP8*) related to cold tolerance were also identified. *TaWCI16* was characterized in wheat and involved in freezing tolerance (Sasaki *et al.* 2013). The locus was identified in environmental association with “minimum temperature of coldest month (BIO6).” *OsiSAP8* is a rice (*Oryza sativa*) locus, which has been associated with cold, drought, and salt stress response (Kanneganti and Gupta 2008). *OsiSAP8* was identified by a SNP in the upper 1% of *F*_ST_ values. While no previously identified drought tolerance loci from barley were detected in our analysis, we find evidence of contributions from three loci previously characterized in three other plant species as contributing to drought tolerance (Table 2). One of these genes was identified based on environmental association while the other eight involved *F*_ST_ comparisons; only two of the SNPs were included in the upper 1%. In the top 2.5% of values, we found an additional six genes previously characterized in two other plant species as contributing to drought tolerance (see Table 2 for details). The identification of multiple characterized loci between the upper 2.5% and 1% of *F*_ST_ is indicative of the trade-off between false discovery and false negative rate in empirical scans for adaptive variation (see Teshima *et al.* 2006) (Table 4).

**Table 4:** The number of SNPs significantly associated with climatic factors and known genes they hit

	Categories	Number	Climatic factors
**Flowering time**	SNPs	3	3, 7, 8, 14, and 17
	Genes	3	—
**Cold tolerance**	SNPs	1	6
	Genes	1	—
**Drought tolerance**	SNPs	1	IC1
	Genes	1	—

Note: Abbreviations for climatic factors are listed under Table 2.

### Relative differentiation among partitions of the sample

Comparison of average *F*_ST_ values across various partitions of the dataset provides a means of determining the factors that contribute most to differentiation in barley landraces. Average *F*_ST_ was highest for the longitude comparison with mean genome-wide *F*_ST_ = 0.123 (± 0.13) (Table S9; Supplemental data 10). A primary partitioning of barley populations by longitude, reported as eastern and western populations, has been reported previously (Morrell and Clegg 2007; Saisho and Purugganan 2007; Poets *et al.* 2015). The second-highest average *F*_ST_ was for elevation at 0.089 (± 0.10) (Table S9; Supplemental data 10). The three-level comparison of *F*_ST_ from 0 - 1,500 m, 1,501 - 3,000 m, and >3,000 m resulted in a slightly lower average *F*_ST_ = 0.0826 (± 0.0745) (Table S9; Figure S7; Supplemental data 10). A three-level comparison of latitude with comparisons above and below the range of wild barley (see Materials & Methods for details) was similar to elevation with average *F*_ST_ = 0.087 (±0.082) (Table S9; Figure S7; Supplemental data 10). Pairwise comparisons of the wild range to high latitude, wild to low latitude, and plant growth habit as either spring or winter barley resulted in much lower average *F*_ST_ values (Table S9).

### F_ST_ outliers from geographic patterns and growth habit

We focused on comparisons most directly linked to climatic differentiation in *F_ST_* outliers. We obtained results from the two-level comparisons for high and low latitude, elevation, and growth habit. The upper 1% of *F*_ST_ values from each comparison yielded 55 outlier SNPs for a total of 203 unique SNPs (Figure 1; Figure 2; Table S11). The comparisons tend to identify unique SNPs. There is an overlap of four SNPs in the low and high latitude comparisons and seven SNPs between elevation and growth habit, but other overlaps were not detected (Figure S8). Winter barleys are less frequently grown at higher latitudes and elevations due to harsh winter weather conditions, and indeed winter barleys from these locations are relatively uncommon in the sample, thus constraining the comparisons (Table S1). The elevation comparison identified the largest number of previously characterized loci including *HvPhyC*, *HvICE2*, and *OSiSAP8* (Figure 2).

**Figure 3:**
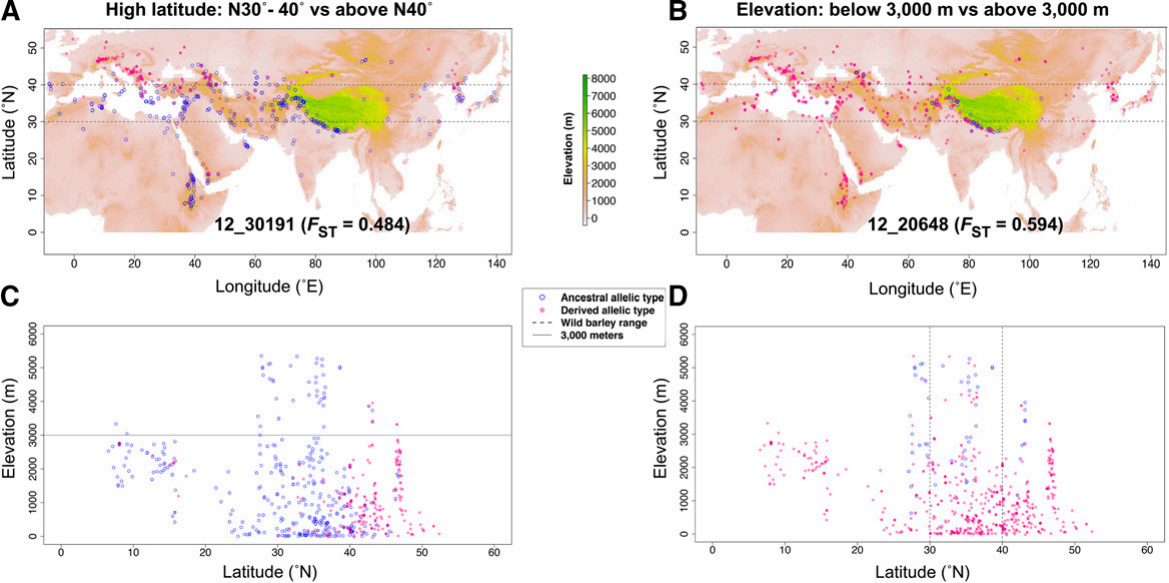
The geographic distribution of the SNPs with high *F*_ST_. (A & C) The geographic distribution of allelic types of 9K SNP 12_30191 with highest *F*_ST_ = 0.4839. The *F*_ST_ was from the high latitude (HL) comparison. (B & D) The geographic distribution of allelic types of 9K SNP 12_11529 with highest *F*_ST_ value of 0.6493. The *F*_ST_ was from the elevation comparison. The color bar indicates the elevations in meters. The filled pink circles indicate the derived allele, while the blue open circles indicate the ancestral allele.

SNPs with the most extreme *F*_ST_ values for elevation, growth habit, and latitude comparisons formed distinct geographic patterns. Each comparison with the highest *F*_ST_ values occurred with SNPs that fall within genes that are annotated, but uncharacterized. The highest *F*_ST_ from the high latitude comparison occurred at SNP 12_30191 with *F*_ST_ = 0.484 (*p*-value = 0). The ancestral allele dominates within the wild barley geographic range for this SNP. Whereas the derived allele is more prevalent in higher latitude regions including the northern extent of the range of wild barley. This range extends across Eurasia from the Central Iberian Peninsula to the Northern Japanese Archipelago (Figure 3A; Supplemental data 10). The highest *F*_ST_ from the low latitude comparison is for the SNP SCRI_RS_153793 with *F*_ST_ = 0.505 (*p*-value = 0). The ancestral state for the SNP predominates within the geographic range of wild barley and higher latitudes. Whereas the derived allele is more prevalent in lower latitudes, which includes the southern extent of the range of wild barley. For landraces, this extends from northwestern Africa to just south of the Japanese Archipelago (Figure S9A; Supplemental data 10). The highest *F*_ST_ between samples from the elevation comparison is for SNP 12_20648 with *F*_ST_ = 0.594 (*p*-value = 0). This SNP’s ancestral allele occurs at high elevations, such as the Himalayan Mountains, while the derived allele tends to occur at lower elevation (Figure 3B; Supplemental data 10). The highest *F*_ST_ between samples from the growth habit comparison was for SNP SCRI_RS_134850 with *F*_ST_ = 0.390 (*p*-value = 0) (Figure S9B; Supplemental data 10). The ancestral SNP state is C, and the derived state is T. The CC genotype is observed in 60.5% of winter barley while TT is observed in 88.2% of spring barley (Figure S9B; Supplemental data 7).

### Environmental association to bioclimatic variables

Using a mixed linear model, we identified 155 unique SNPs significantly associated with at least one environmental factor with the threshold of FDR < 0.25 (Figure 1; Table S8). The first three PCs explained (48.07%) of the variance (Figure S1) and were used to control for population structure. All of the *p*-values and Benjamini-Hochberg FDR-values were reported in Supplemental data 9.

We found 81 SNPs associated with precipitation (variables BIO12 to BIO19) and 51 with temperature (all variables from BIO1 to BIO11) for individual environmental variables (Figure 1). We also identified 47 SNPs associated with geographic variables (latitude, longitude, and elevation), and 32 associated with independent components (top three independent components calculated from BIO1 to BIO19 values after standardization for each BIO variable, called ICs) (Figure 1). Another finding includes 47 cases where individual SNPs were associated with more than one environmental variable (Figure S10). But more generally, as with the *F*_ST_ comparisons, the environmental variables tend to associate with unique sets of SNPs (Figure S10). The largest proportion of unique SNPs were found for precipitation (33.55%), followed by geographic variables (18.71%), temperature variables (18.06%), and then ICs (1.29%) (Figure S10). The aggregated independent components generally did not identify novel variants.

### Minor allele frequency of identified SNPs

The SNPs identified as *F*_ST_ outliers have average MAF = 0.330 (± 0.101) *vs.* a sample-wide average of 0.262 (± 0.140) a highly significant difference (Mann-Whitney U Test, *p*-value = 3.5 × 10^−15^). SNPs with significant environmental associations have an average MAF = 0.251 (± 0.137), which is slightly lower than the full SNP data set but not statistically significant (Mann-Whitney U Test, *p*-value = 0.3114). While MAF limits the potential to associate genotype to phenotype for association analysis; the relatively large sample represented here does not suffer from this major limitation of detection. The high MAF contrasts with expectations that adaptive variants for less frequently occupied habitats, such as high elevation sites of cultivation, should be relatively uncommon (average MAF = 0.240 (± 0.091) of outliers from elevation comparison). A relatively low MAF might be expected under models where adaptive variants in a particular environment exhibit antagonistic pleiotropy, and thus confer lower fitness away from habitats in which they are adaptive (Tiffin and Ross-Ibarra 2014).

### SNP density and LD Near focal SNPs

**Figure 4:**
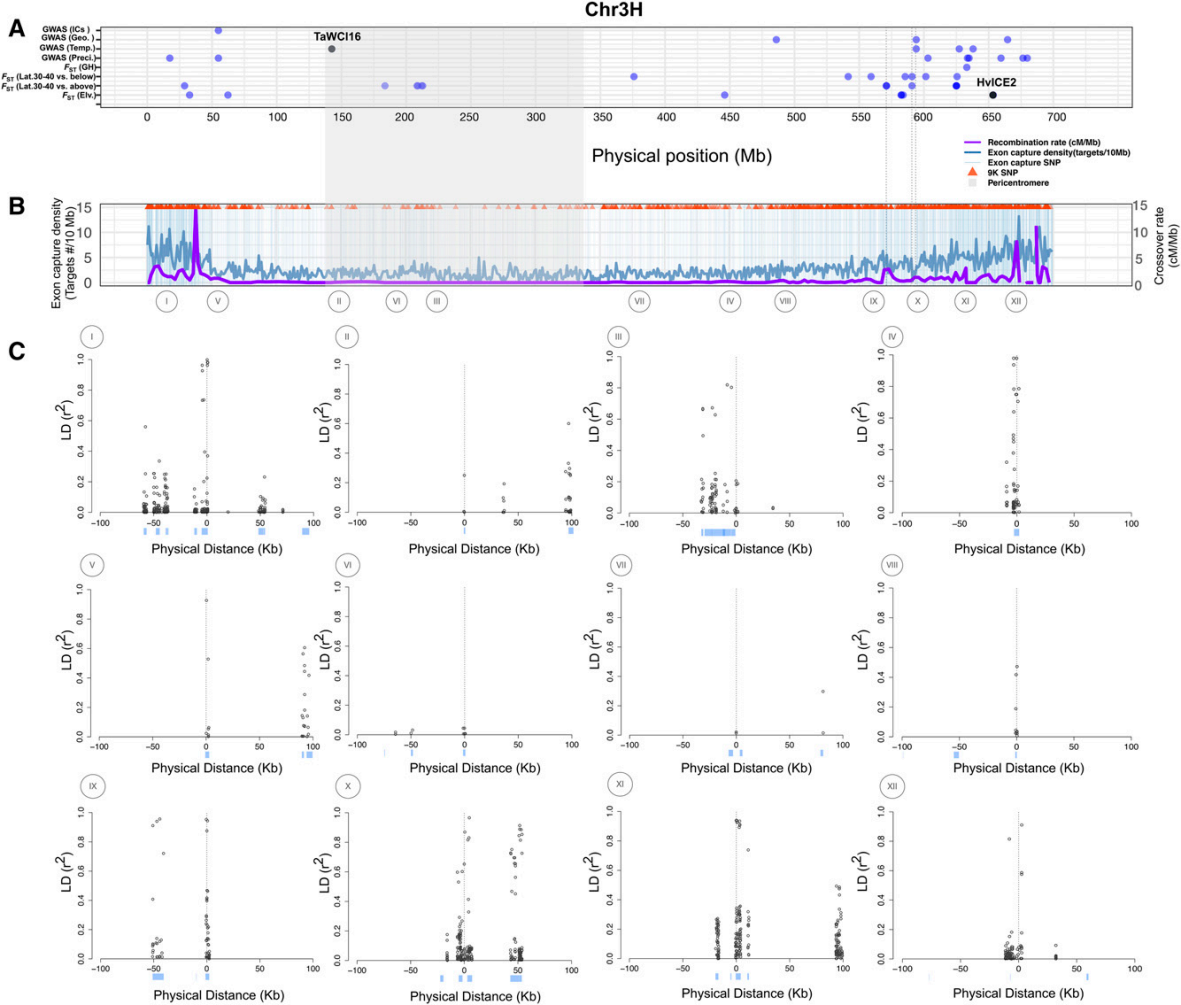
(A) The genomic distribution of outlier SNPs identified according to the *F*_ST_ comparisons of elevation (below 3,000 m *vs.* above 3,000 m), low latitude (below 30°N *vs.* 30-40°N), high latitude (30-40°N *vs.* above 40°N), growth habit (winter *vs.* spring) and association analysis of 21 bioclimatic variables, which are categorized into three classes (precipitation, temperature, and geographic variables) on chromosome 3H. (B) Exome capture target density (dark blue line), crossover rate in cM/Mb (purple line), the genomic distribution of SNPs identified in the 62 barley landraces (vertical light blue lines), and 9K SNPs (red triangles) on chromosome 3H. The vertical dotted lines in panels (A) and (B) indicate that those outlier SNPs are shared across different traits. (C) LD plots for SNPs significantly associated with at least one bioclimatic variable (bottom) on chromosome 3H. Each plot shows a 200 Kb window, 100 Kb on either side of the SNP. For the LD plots, genotyped SNPs are at location 0, and positions upstream and downstream are listed as negative and positive values. The light blue bars are genes in 200 Kb windows surrounding the genotyped SNPs. The SNPs from the I to XII are: 11_20742, 11_10380, SCRI_RS_173916, 12_20108, 11_10601, 12_31008, SCRI_RS_173717, SCRI_RS_6793, SCRI_RS_207408, 12_10210, SCRI_RS_192360, and 12_30960.

As previously reported, SNP density is highest on chromosome arms and lower in pericentromeric regions (Muñoz-Amatriaín *et al.* 2015; Mascher *et al.* 2017). This trend is particularly evident for 9K SNPs (Figure 4B; and Figure S3) and is broadly consistent with lower SNP density in genomic regions with lower observed rates of crossover (Muñoz-Amatriaín *et al.* 2011). Exome capture density is also lower in pericentromeric regions, such that 51,567 SNPs are detected in 1.560 Gb in pericentromeric regions (33 SNPs/Mb) *vs.* 431,147 SNPs in 3.02 Gb (143 SNPs/Mb) on chromosome arms.

We compared LD at queried SNPs to the surrounding region for 358 SNPs identified by environmental association or as *F_ST_* outliers. We observed 89.3% of these SNPs in exome capture resequencing. The remaining 10.7% of the queried SNPs were replaced by proximal SNPs with similar MAF. The replaced SNPs had an average MAF of 0.035 (± 0.005) and were on average 32.9 Kb (± 31.5 Kb) from the physical position of the queried SNPs (Figure S11). For 123 (34.4%) SNPs, LD with *r*^2^ > 0.45 (90^th^ percentile) occurred within the same gene (Figure S12A & B; Table 5). Detectable LD with flanking loci is limited in pericentromeric regions because the locus tested is often the only annotated gene within the 200 Kb window (Figure 4C). For an additional 212 (59.2%) SNPs, LD extends well beyond the locus where the initial association was identified (Figure S12 C & D). For 23 SNPs (6.4%) there was either no LD with the focal SNPs or no SNPs identified in the 200 Kb window around the focal SNP (Table 5). These results indicate that the potential to identify individual loci that contribute to adaptive phenotypes is impacted by recombination rate variation and gene density across the genome (Figure 4B, Figure S3).

**Table 5:** Linkage disequilibrium (LD) for all SNPs associated with environmental variables or identified as *F*_ST_ outliers

	LD within a gene	Extended LD	No LD	Missing*
*F*_ST_	61	132	10	0
**Association**	62	80	7	6

Note: * indicates that there are no SNPs in the 200 Kb window around the target SNP.

### Putative structural variation

An examination of *F*_ST_ outliers prior to LD filtering identified 15 SNPs with *F*_ST_ of ∼0.40 for the elevation comparisons. All occur on chromosome 5H at 663.25 cM based on the consensus genetic map (Muñoz-Amatriaín *et al.* 2011). These SNPs span a physical distance of 133.7 Mb (Table S12). The minor allele frequencies of these SNPs are very similar (0.354 - 0.361) as expected based on *F_ST_* values, with minor alleles occurring in the same individuals in almost all cases. All 15 SNPs are in nearly complete LD. The region that contains the SNPs is between 131.2 Mb and 265.0 Mb of chromosome 5H and overlaps with a region identified as a putative chromosomal inversion in wild barley (Fang *et al.* 2014). The SNPs that Fang *et al.* (2014) associated with the putative inversion occur between 126.7 Mb and 305.5 Mb. Evidence for an inversion in wild barley was based on elevated *F*_ST_ values, extended LD, and enrichment for environmental associations. Fang *et al.* (2014) reported a similar pattern on chromosome 2H in wild barley at positions that correspond to 267.3 Mb to 508.7 Mb. We found less evidence of allele frequency differentiation on 2H than in wild barley; observing two SNPs which span ∼494 Kb with *F*_ST_ = 0.33 in our sample of landraces (Table S12).

### Haplotype analysis at individual genes

**Figure 5:**
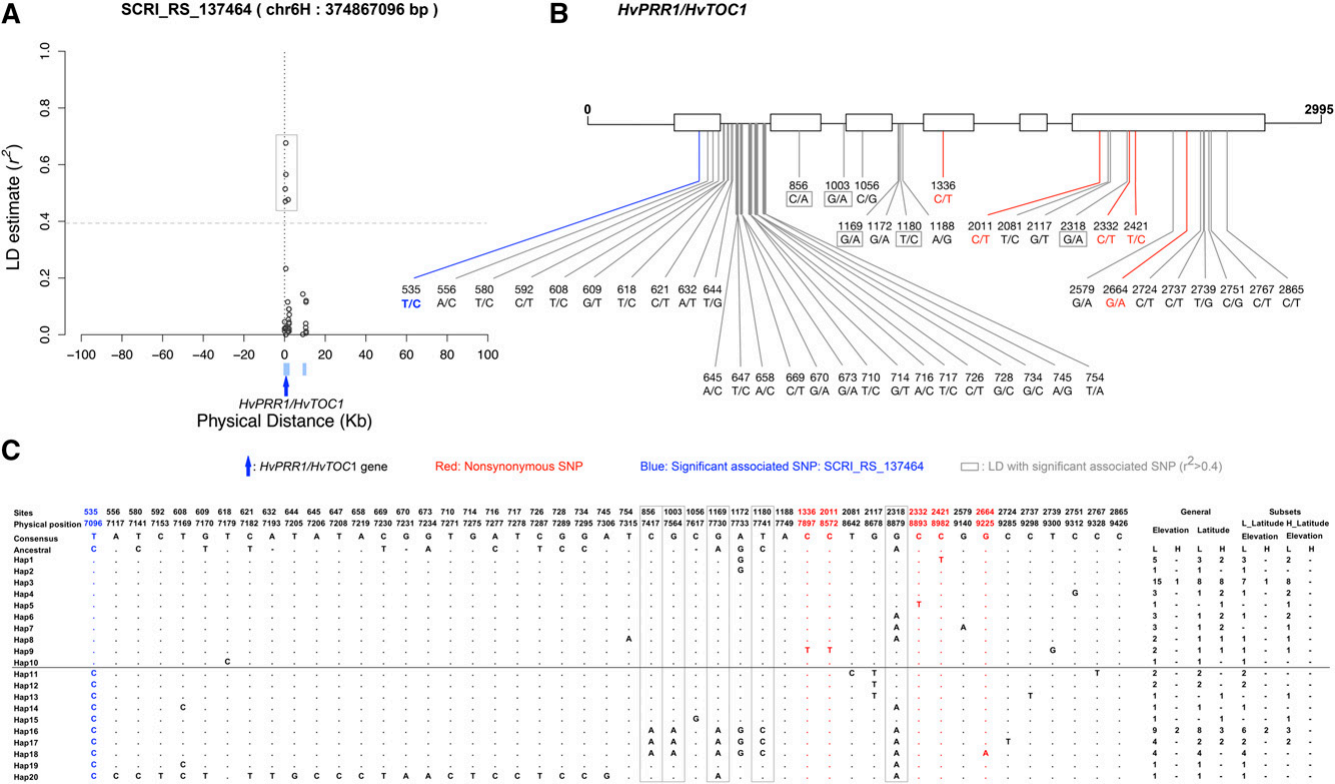
(A) The linkage disequilibrium (LD) analysis of genotyped SNP SCRI_RS_137464, which is significantly associated with BIO8 (“mean temperature of the wettest quarter”). The blue bars indicate genes in the 200 Kb window surrounding SCRI_RS_137464, the red arrow indicates the *HvPRR1/HvTOC1* (flowering time related gene) that includes SNP SCRI_RS_137464 (B) The gene structure of *HvPRR1*/*HvTOC1* and the functional annotation of SNPs in this gene. (C) Haplotype structure of *HvPRR1*/*HvTOC1* based on the SNPs in this gene. L: low; H: high.

Environmental association results identified a SNP, SCRI_RS_137464, significantly associated with mean “temperature of wettest quarter (BIO8)” (*p*-value = 8.56 × 10^−4^), which is in the *HvPRR1/HvTOC1* gene (Figure 5A). *TOC1* is an important component of the circadian clock in *Arabidopsis*. It conveys a crucial function in the integration of light signals to control circadian and morphogenic responses, which is closely related to flowering time (Más *et al.* 2003). *HvPRR1/HvTOC1* is the ortholog of *TOC1* in *Arabidopsis thaliana* and has a high level of sequence similarity and conservation of diurnal and circadian expression patterns when compared to *TOC1* in *Arabidopsis* (Campoli *et al.* 2012). Exome capture resequencing data identified 48 SNPs including SCRI_RS_137464 in *HvPRR1/HvTOC1*. Five SNPs at the locus annotate as nonsynonymous and are the most obvious candidates to contribute to functional variation. Four of these are in the last exon of the gene (Figure 5B & C). Five SNPs within *HvPRR1/HvTOC1* have relatively strong LD with SCRI_RS_137464 (*r*^2^ > 0.45) (Figure 5 A & B). Resequencing identified 20 haplotypes with no obvious geographic pattern (Figure 5C).

Environmental association of both “temperature of coldest month (BIO6)” and “mean temperature of the coldest quarter (BIO11)” identified an association on chromosome 3H with SNP 11_10380 (*p*-value = 4.95 × 10^−4^). The SNP is in the barley gene *HORVU3Hr1G030150.1*, which is an ortholog of the wheat gene *WCI16* (*Wheat Cold Induced 16*) (Sasaki *et al.* 2013) (Figure S13). The derived alleles for genotyped SNPs at this locus are much more common in landrace barley than in wild lines. In previous published wild barley genotyping data (Fang *et al.* 2014) the minor allele at 11_10380 occurs in four accessions with geographic provenance information. Those accessions occur at an average of 1,460 m - near the upper end of the elevational range for wild barley. Estimated derived allele frequencies differ considerably in wild barley and landraces, at 0.0072 and 0.13 respectively. The 200 Kb window surrounding the SNP contains one gene in addition to *HvWCI16* (Figure S13A). *TaWCI16* encodes a putative transcription factor involved in stomata development. It represents a novel class of late embryogenesis abundant (LEA) proteins in response to cellular dehydration and is involved in freezing tolerance (Sasaki *et al.* 2013). *TaWCI16* was shown to improve freezing and cold temperature tolerance in wheat when transformed into *Arabidopsis thaliana* (Sasaki *et al.* 2013). There were six SNPs identified using exome capture sequencing from 61 landraces which includes 11_10380. Three of six SNPs, including 11_10380, are in noncoding sequence (Figure S13B). Of the three SNPs observed in coding regions, one is a nonsynonymous change at nucleotide position 119. This changes valine to leucine, which have similar properties. There is no evidence of LD between this SNP and others within a 200 Kb window (Figure S13A). Exome capture resequencing identified eight haplotypes, with three of the five being relatively common. Seven haplotypes predominate at lower elevation and lower latitude, with two of those occurring most frequently (Figure S13C).

Environmental association analysis suggested that the SNP SCRI_RS_235243 significantly (*p*-value = 3.62 × 10^−4^) associated with “precipitation of driest months (BIO14)” hit the barley gene *HORVU1Hr1G008120.1*. This is an ortholog that produces dehydroascorbate reductase (*DHAR*; EC 1.8.5.1) in *Arabidopsis thaliana* and bread wheat. It is one of two important enzymes functioning in the regeneration of ascorbate (AsA) which plays a role in protection against oxidative stress (Eltayeb *et al.* 2006; Osipova *et al.* 2011). The 200 Kb window surrounding the genotyped SNP SCRI_RS_235243 contains four genes in addition to *DHAR*, which includes two with exome capture sequence coverage (Figure S14A). Previous results suggest that overexpression of *DHAR* can protect plants against drought, salt, and polyethylene glycol-induced stress in tobacco and bread wheat (Eltayeb *et al.* 2006; Osipova *et al.* 2011). Resequencing identified 53 SNPs in our panel, including SCRI_RS_235243. This encompassed 28 SNPs in noncoding regions, 14 synonymous, and 11 nonsynonymous (Figure S14 B & C). SCRI_RS_235243 is one of nine nonsynonymous SNPs in the first exon of the *DHAR* gene (Figure S14 B & C). Six SNPs are in high LD with SCRI_RS_235243 (*r*^2^ > 0.45), all are noncoding variants within *DHAR* (Figure S14 B & C). The derived variant at SCRI_RS_235243 occurs within two haplotypes (Figure S14C) that occur in high latitude regions.

A putative causative variant is not immediately apparent for all three of the loci described. However, as the loci *HvPRR1/HvTOC1* and *DHAR* demonstrate, barley landraces are frequently segregating for an abundance of potentially functional variants.

## Discussion

Examination of environmental associations to bioclimatic variables and allele frequency outliers in a broad collection of Old-World barley landraces has identified six loci with prior evidence of contribution to climatic adaptation in barley (Table 1; Table 2). This includes well-characterized loci that contribute to flowering time, cold, or drought adaptation in barley including *HvCbf4B* (Stockinger *et al.* 2007), *HvICE2* (Skinner *et al.* 2006), *PhyC* (Nishida *et al.* 2013), *HvPpd-H1 (HvPRR37)* (Turner *et al.* 2005; Jones *et al.* 2008), and *HvVrn-H1* (*HvAP1*) (Cockram *et al.* 2007). All of these loci have been shown to alter phenotypes that are potentially associated with adaptation across the very broad geographic range of cultivation.

### Orthologs that potentially played an adaptive role

We found six loci as *F*_ST_ outliers or in environmental associations that had previously been identified as contributing to flowering time, cold, or drought stress in other plant species (Table 2). This includes one flowering time-related locus characterized in *Arabidopsis thaliana*, *AtCOP1* (Xu *et al.* 2016), which was identified as photomorphogenic repressors and regulates flowering time (Lau and Deng 2012). Two loci related to cold tolerance were identified. This included wheat locus *TaWCI16* involved in freezing tolerance (Sasaki *et al.* 2013) and the rice (*Oryza sativa*) locus *OsiSAP8* which has been associated with cold, drought, and salt stress response (Kanneganti and Gupta 2008). *TaWCI16* was induced during cold acclimation in winter wheat (Sasaki *et al.* 2013). *OsiSAP8* can be induced by multiple-stresses including heat, cold, salt, desiccation, submergence, wounding, heavy metals, and the stress hormone abscisic acid (Kanneganti and Gupta 2008). For drought tolerance, we identified nine orthologs characterized in five other plant species (Table 2). For example, over-expression of the wheat expansin gene *TaEXPA2* improved seed production and drought tolerance in transgenic tobacco plants (Chen *et al.* 2016).

### Why did previously identified genes go undetected in our study?

The genetic basis of flowering time in barley has been explored extensively and multiple genes have been cloned (see Hansson *et al.* 2018). However, relatively few cold or drought tolerance related genes have been characterized or cloned (Visioni *et al.* 2013; Honsdorf *et al.* 2014). Based on a literature search, we identified 57 flowering time and 33 cold tolerance related genes in barley (Table S5; Table S6). Our analyses found ∼10% of flowering time and ∼12% of cold tolerance related genes (Table 1). We did not identify any of the 13 previously reported drought tolerance related genes (Table 1; Table S7).

Why were more previously identified genes not detected? Not every gene was genotyped by the 9K SNPs and many genes genotyped are represented by a single SNP or a small number of SNPs (Table 1). The surveyed SNPs have an average MAF of 0.30 (± 0.11). As in standard association mapping, the SNPs genotyped need to occur in LD with a causative variant (Balding 2006); even SNPs in close physical proximity can occur on alternate haplotypes and have limited LD (Nordborg and Tavaré 2002). For flowering time, 29 of 57 genes were genotyped by at least one SNP. Nine genes were genotyped by two or more SNPs (Table 1). All but one of these genes, *HvVrn-H3/HvFT1*, was found in our analysis (Table S13). All nine of these genes have been identified in previous geographic comparisons in barley (Muñoz-Amatriaín *et al.* 2014; Russell *et al.* 2016; Contreras‐Moreira *et al.* 2019). Among genes identified by multiple SNPs is *HvPpd-H1/HvPRR37*, a key regulator of flowering (Turner *et al.* 2005; Jones *et al.* 2008). This gene was genotyped by eight SNPs with five SNPs identified as outliers in our comparisons. Previous studies have identified the *HvCEN* and *HvVrn-H2/HvZCCT-Ha/b/c* genes associated with flowering time as allele frequency outliers (Muñoz-Amatriaín *et al.* 2014; Russell *et al.* 2016; Contreras‐Moreira *et al.* 2019). *HvCEN* was not genotyped by any SNP in our panel. *HvVrn-H2* was genotyped by a single SNP with an *F*_ST_ = 0.143 in the elevation comparison, at the 75^th^ percentile in this comparison and thus below the 99^th^ percentile threshold. In summary, genes identified through top-down approaches are generally identified in our comparison if they are represented by a sufficient number of SNPs (Table S13). Genes can contribute to phenotypic variation without having played a role in previous rounds of adaptation (Ross-Ibarra *et al.* 2007; Kantar *et al.* 2017). However, given current SNP densities, it is premature to conclude that any of the absent loci did not contribute to adaptation in barley.

### Comparison to previous studies

Three of the loci we identified as contributing to adaptive differentiation in Old World landraces were previously reported as *F*_ST_ outliers. They contribute to geographic differentiation in barley breeding populations in North America (Poets *et al.* 2016). This included *HvCbf4B*, *HvPpd-H1*, and *HvVrn-H1*. They were also found as an *F*_ST_ outlier and in association with temperature adaptation in comparisons of wild barley populations (Fang *et al.* 2014). Two of the loci we identified as contributing to flowering time and cold tolerance, including *HvPpd-H2/HvFT3* and *HvCbf4*, were also found as outliers in allele frequency in barley landraces in Spain (Contreras‐Moreira *et al.* 2019). However, we did not identify other loci that overlapped between our study and Contreras‐Moreira *et al.* (2019).

We focused on SNP comparisons, but also found evidence that a large chromosomal inversion has contributed to elevational adaptation in barley. On chromosome 5H, 15 SNPs have *F*_ST_ of ∼0.40 in the elevation comparison. All occur at the consensus genetic map position of 663.25 cM, which is consistent with the regions reported in barley landraces from Spain (Contreras‐Moreira *et al.* 2019). Fang *et al.* (2014) characterized the region as a putative chromosomal inversion that differs in frequency between the eastern and western portions of the geographic range of wild barley. Recent studies have identified putative chromosomal inversions that contribute to elevation and temperature gradients in teosinte and maize (Fang *et al.* 2012; Hufford *et al.* 2012; Pyhäjärvi *et al.* 2013), rainfall regime and annual *vs.* perennial growth habit in *Mimulus guttattus* (Sweigart and Willis 2003; Lowry and Willis 2010; Twyford and Friedman 2015), and temperature and precipitation differences in wild barley (Fang *et al.* 2014). In a close parallel to our results, an inversion on maize chromosome 3 appears to contribute to teosinte adaptation to high elevation and also has impacted highland adaption in maize through altered flowering time (Wang *et al.* 2017).

### Advantages of study design and future prospects

SNP density is a limitation in our study. With ∼40,000 annotated genes in the barley genome (Mascher *et al.* 2017), roughly one in six genes was directly genotyped. For about one-third of SNPs, there is limited LD with nearby loci (Figure 4; Figure S12). In regions of the genome with high crossover rates and higher gene density, LD can be limited beyond the locus containing the genotyped SNP (Figure 4C and Figure S3). In regions with limited crossover, gene density is also low (Figure 4B and Figure S3). LD would typically have to extend hundreds of kilobases between genotyped SNPs and a causative variant at another locus (Figure S12) to create an association. High MAF of genotyped variants may also contribute to limited LD. Common variants are typically older and have experienced more recombination, and can be closer to linkage equilibrium (Nordborg and Tavaré 2002).

Our study benefits from large sample size. Russell *et al.* (2016) performed environmental association with 1,688,807 SNPs from exome capture resequencing in 137 cultivated samples. While the analysis identified 10 loci associated with flowering time, many other previously reported genes went undetected. This prompted the authors to suggest a lack of power owing to small sample size (Russell *et al.* 2016). Population-level sampling of individual landraces would make it possible to use more powerful allele frequency-based approaches such as those of Coop *et al.* (2010), for detecting local adaptation. Landraces of many crops are relatively heterogeneous (Brown 2000), and species such as barley maintain relatively high levels of diversity within landrace populations (Rodriguez *et al.* 2012).

Despite limited SNP density and the sampling of relatively common variants, our comparative analyses identified a number of previously identified barley loci and many plausible candidate loci from other plant species. Better coverage of barley gene space through exome capture or whole genome resequencing in a relatively deep panel of accessions would likely uncover a much more comprehensive set of variants contributing to environmental adaptation. This could contribute to the targeted use of variants for adaptation to environmental and climatic conditions for barley breeding and germplasm improvement, with the potential to improve the understanding of loci that contribute to climatic adaptation in wheat and other cereals.
